# Identifying subtypes of suicidality: a second larger consensus study in emergency clinical psychiatric practice

**DOI:** 10.3389/fpsyt.2026.1751407

**Published:** 2026-03-05

**Authors:** Remco F.P. de Winter, Damien S.E. Broekharst, Connie M. Meijer, Nienke Kool-Goudzwaard, Anne T. van den Bos, John H. Enterman, Manuela Gemen, Chani Nuij, Mirjam C. Hazewinkel, Danielle Steentjes, Gabrielle E. van Son, Jonas G. Weijers, Derek P. de Beurs, Marieke H. de Groot

**Affiliations:** 1 School for Mental Health and Neuroscience (MHeNs), Maastricht University, Maastricht, Netherlands; 2 Department Suicide Prevention in Mental Health (SuPreMH), Mental Health institute Rivierduinen, Leiden, Netherlands; 3 Psychiatry Department, Mental Health Institute Parnassia Group, The Hague, Netherlands; 4 Section of Clinical Psychology, Free University (VU), Amsterdam, Netherlands; 5 Section of Psychology, Health and Technology, University of Twente, Enschede, Netherlands; 6 Psychology Department, University of Amsterdam, Amsterdam, Netherlands; 7 Psychiatry Department, Mental Health Institute Lentis, Groningen, Netherlands

**Keywords:** differentiation of suicidality, suicidal subtypes, suicidal typology, SUICIDI, perceptual disintegration, primary depressive cognition, psychosocial turmoil, inadequate coping/communication

## Abstract

**Background:**

A model has been developed to distinguish subtypes of pathways to entrapment leading to suicidality in clinical mental health practice. The (hypothetical) 4-type Model of Entrapment ((h)4ME) delineates four subtypes of suicidality: I) Perceptual Disintegration (PD), II) Primary Depressive Cognition (PDC), III) Psychosocial Turmoil (PT) and IV) Inadequate Coping/Communication (IC).

**Objective:**

To examine the model’s usability and feasibility in a larger cohort of suicidal patients (n= 75) following a pilot study.

**Methods:**

Consultation reports to general practitioners of 75 suicidal emergency patients were independently allocated to subtypes by three psychiatrists and three nurses using the SUICIDI-3 tool. This tool describes the proposed subtypes. Interrater agreement was assessed by calculating Intraclass Correlation Coefficients (ICCs). Absolute and dimensional type agreement was established to assess the model’s usability and the SUICIDI-3 tools feasibility.

**Results:**

All raters were able to assign cases to subtypes. Excellent absolute Type Agreement (aTA) was observed for PD (0.96) and PDC (0.92), and good aTA for PT (0.83) and IC (0.83). For dimensional Type Agreement (dTA) the ICC was excellent for PD (0.97), PDC (0.95) and IC 0.92), and good for PT (0.88).

**Conclusions:**

The (h)4ME demonstrates promising usability and feasibility when tested by staff of psychiatric emergency services. Replication studies of samples of various clinical, demographic or ethnic origin and of diverse professional background and contexts are needed to confirm the consistency of these findings.

## 1 Introduction

Suicidality is a serious mental health problem and plays a significant role in clinical practice. For about one-third of the patients assessed by Dutch psychiatric emergency outreach services the main reason for consultation is suicidality (1, 2). Suicide and suicide attempts represent the worst outcome in mental health care, affecting patients, their relatives, mental health care workers and other caregivers (3).

Reliable prognostic models for attempted or completed suicide are not available (4). Although previous suicide attempts are strongly associated with completed suicide, attempts are not always related to the severity of observed symptoms (5). Suicide-risk factors are routinely addressed during assessment, but the outcome of risk assessment remains uncertain and may lead to defensive or even iatrogenic actions (6).

Generally, it can be stated that medical knowledge of disorders has increased once differentiation of clinical pictures becomes possible. For example, the distinction between small-cell and non-small-cell bronchial carcinoma is crucial for treatment and prognosis (7). The current understanding of suicidality, however, has not yet reached the level of full comprehension regarding the mechanisms leading to suicidal conditions. Suicidality caused by a severe loss differs from suicidality arising from psychosis in which the patient hears voices that instruct them to end their life. However, no clear model has been developed to describe this distinction. Despite the availability of previous subtyping of suicidality, a model which has achieved a level of usability in clinical practice is lacking (8).

We propose that distinguishing pathways leading to suicidality may provide clinicians with a firmer grasp of this complex, heterogeneous, and multilayered phenomenon. It may allow the application of tailored assessment and treatment strategies, and a better estimation of responsibilities of mental health care professionals and patients to prevent suicidal acts (9–11). Differentiation may also facilitate targeted research into the effectiveness of various suicide prevention strategies, allowing identification of effective specific type approaches (12). Furthermore, a common language among care providers, both within and outside mental health care, to describe different forms of suicidality may facilitate a better understanding of of patient-specific needs.

**Figure 1:**
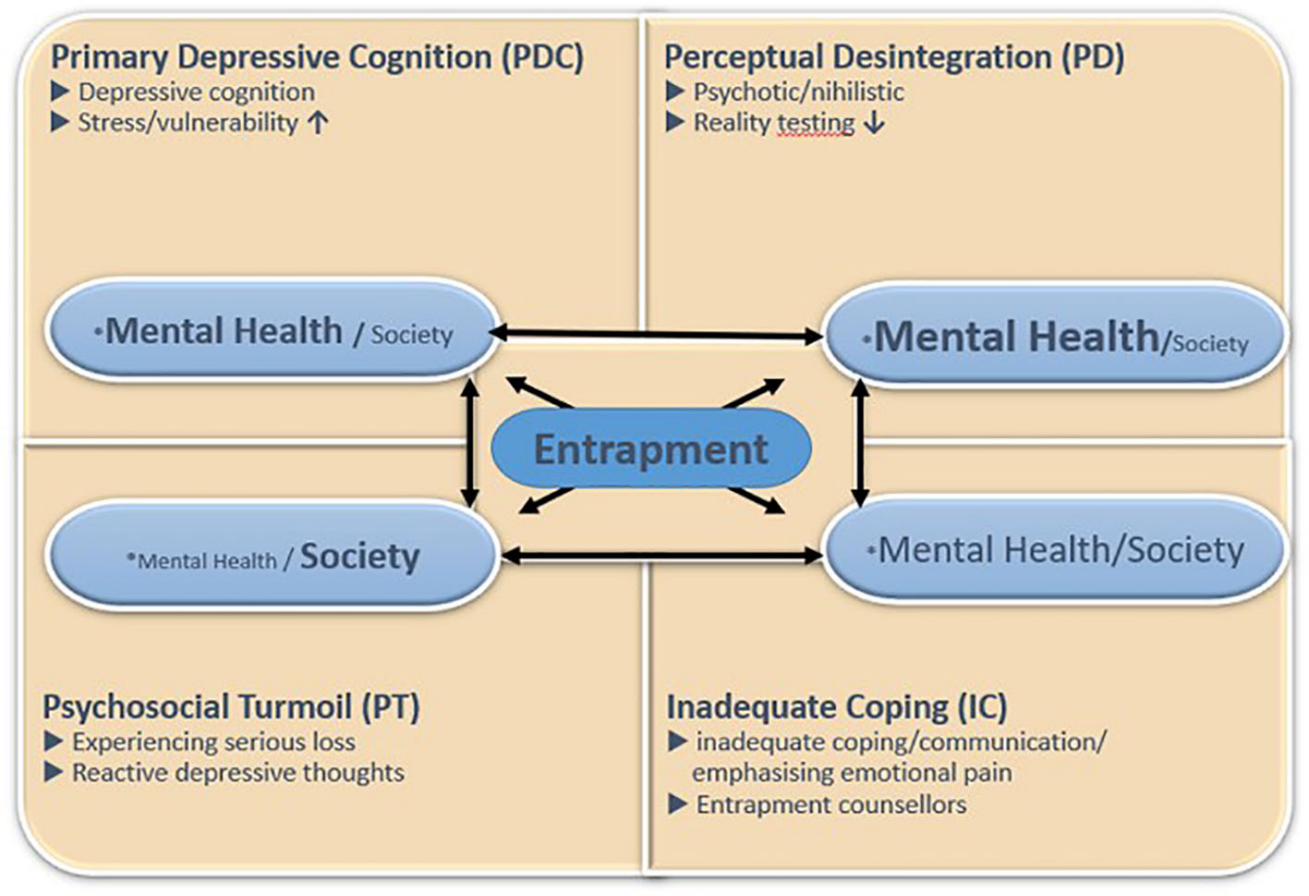
The hypothesized four-type model of entrapment of suicidality including the degree of responsibility for (mental) health care, patient or the community (as described in De Winter et al., 2023 (15)). *The emphasis on who (society and/or mental health) may be responsible for preventing suicidal actions per subtype. The relative size of the *Mental Health* and *Society* elements reflects the prominence of mental health–related and societal/cultural influences, while arrows indicate dynamic interactions between subtypes.

Based on clinical experience and practice a model has been developed to describe subtypes of suicidality (12, 13); the (hypothetical) 4-type Model of Entrapment ((h)4ME). Entrapment is a state of mind in which a person believes there is no other way to escape from it than by taking their own life (14). The model (**Figure 1**) (13), distinguishes four types of pathways leading to entrapment: Perceptual Disintegration (PD), II: Primary Depressive Cognition (PDC), III: Psychosocial Turmoil (PT), and IV: Inadequate Coping/Communication (IC). The subtypes have been described in detail elsewhere (12, 13, 15), see also **Box 1**; **Figure 1**.

Box 1 **Descriptions of the four subtypes of suicidality. (previously presented as Table 1, (15)**

Perceptual Disintegration (PD)

Suicidality originates foremost in psychosis, which can often be accompanied by affective (depressive) dysregulation or can be affected by it. Usually, the psychotic state has only been present for a short period of time (days or weeks rather than months) and is noticed (or becomes apparent) due to its severity. Suicidality may originate in the cognition; in which case, however, the severity has developed to such a level that it can be considered as a mood-congruent or mood-incongruent psychotic state. The distress can be understood, but the severity and suffering can no longer be perceived as comprehensible by the examiner. A classic state is suicidality during a depression with mood-congruent psychotic features. However, it can also appear in people who, while in a psychotic state, are ordered by their delusions and/or hallucinations to hurt themselves.

Primary Depressive Cognition (PDC)

Suicidality stems primarily from a depressive thought process and there are no psychotic features (yet). It is possible that the depressive state is present for a period of time(weeks or months). Thoughts of suicide, which are part of the cognition are characteristic and are present on a daily basis. There is clear evidence of distress, which can be noticed by the examiner due to the depressive thought process. A classic example is a depressive disorder, but primary depressive cognition may also be part of an anxiety disorder, autism spectrum disorder, etc. The thoughts may also be existential without meeting the criteria for a depressive disorder.

The features of a personality disorder may be mixed with the depressive state, or the depressive state may be caused by a personality disorder and become part of a returning thought pattern in which negative cognitions and Beck’s cognitive triad of depression can be present (negative views about oneself, negative views about the world, and negative views about the future).

Psychosocial Turmoil (PT)

Suicidality stems primarily from a severe loss or blow to the ego, leading to a complete upheaval of one’s life. The person can experience enormous guilt, severe shame, is afraid to make eye contact, or experiences despair without being in a psychotic state. There is unbearable anguish, which leads to a need to be freed from the perceived pain or the need to no longer exist, without being able to feel any relief or to escape from the awful misery and/or pending dread. Usually, someone has been in this state for a short period of time (hours, days, or weeks). Substance abuse use may amplify the expression of suicidality in PT. The stress is perceivable for the examiner from the perspective of loss or a blow to the ego and there may be slight psychotic features, but the examiner will still be able to follow the narrative. Underlying dysregulation of impulsivity can worsen the state and increases the risk of a lethal outcome.

Inadequate Coping/communication (IC)

Suicidality stems from a severe feeling of distress and the person is not able to communicate this properly. There is difficulty with formulating an adequate request for help and one seems to be hoping for a solution by demonstrating suicidality. This behaviour usually exists for a longer period of time (months) and fluctuates severely over time. This type of chronic suicidality is often seen as part of a personality disorder such as a borderline personality disorder. Also, substance use, may lead to a greater expression of suicidality in IC. Suicidality can be perceived by others as ‘externalizing’ and fake and can result in aid workers feeling ‘trapped’ in the dynamics. The behaviour can coincide with experiences of loss with which the powerlessness is externalized and not internalized. Often, the support system is exhausted and professionals are viewed as failing. The major risk is for professionals to feel manipulated, and for the person who is assessed to feel misunderstood and not taken seriously, which leads to an amplification of the behaviour, accompanied by an increased risk of suicide. Contrary to how it can be perceived by others, the person is genuinely in distress. Suicide can be used as the ultimate way to communicate about the distress caused by the perceived unfairness or judgment of rejection by the subject. Recognizing and exploring the countertransference and offering help to the underlying motivators of suicidality are especially essential with this subtype.

In a pilot study, high Intraclass Correlation Coefficients (ICC) values for absolute and dimensional Type Agreement were shown. ICC values for absolute Type Agreement (aTA) varied from 0.82 to 0.91 and for dimensional Type Agreement (dTA) from 0.79 to 0.93 (15).

The current study re-examined the feasibility and usability of the (h)4ME and the SUICIDI-3.2 tool to achieve further evidence for the model’s reliability and feasibility and the SUICIDI-3.2 tool’s usability. We hypothesize that in a larger cohort of suicidal patients comparable degrees of interrater agreement will be demonstrated as compared to the pilot study (15).

## 2 Methods

### 2.1 Study design and participants

The study used a mixed-methods exploratory design. Expert mental health care professionals (raters, n = 6) assigned anonymized case reports of suicidal patients (n = 75) to one or more subtypes of the (h)4ME using the SUICIDI-tool version 3.2 (15).

Raters comprised three psychiatrists and three mental health nurses recruited from four large mental health care institutions across the Netherlands and England (each employing >2000 staff) (13, 15). All raters were experienced clinicians with more than 15 years of practice and scientific involvement in suicide risk assessment and were employed at the emergency service of the participating institutions. Raters followed a two-hour online training session on the (h)4ME and the use of SUICIDI version 3.2.

Cases (n = 75) were derived from anonymized consultation reports of suicidal patients who were previously assessed by the Hague Psychiatric Emergency Service (13, 15). These case reports with a standardized format were sent to the patients’ general practitioner following the assessments. To obtain consensus, all cases were discussed and evaluated during the psychiatric emergency service team’s morning handover, attended by at least five mental health professionals; they were independent of the raters (13, 15). Cases were included only after receiving informed consent to share anonymized consultation letters with the raters. Overall, 503 consultation letters were recorded in a secured database. Cases were numbered in the order of examination. Cases 1–25 were included in a pilot study (15). Cases 26 up to 100 were considered for the current study. Patient demographics and primary DSM-5 classifications were recorded (16) to verify associations between type assignments and primary diagnoses.

### 2.2 Measurement

Initially, raters were instructed to select one subtype they considered most appropriate, representing the absolute Type Agreement(aTA) score. To assess overlap of subtypes, raters were subsequently asked to distribute a total of four points over various subtypes to yield dimensional Type Agreement(dTA). This was carried out by using the SUICIDI (3.2)-tool designed to enable users to distinguish the subtypes both absolutely (categorically) and dimensionally. The pilot study showed that the four subtypes could be distinguished by using the SUICIDI-3.2 (15). Based on post-pilot study discussions with raters, it was observed that substance use had not been uniformly evaluated across raters, necessitating further refinement of subtype criteria for the current study. It was clear that a thorough explanation to raters of the role of substance use in the etiology of entrapment is important to improve consistency and reliability of the findings. Substance abuse can cause increased impulsivity and may strengthen negative thoughts; this may trigger entrapment (21). However, the use of psychedelics is not associated with an increased risk of suicidality (22). Therefore, in cases involving substance abuse, it is important to investigate how entrapment arose, but not what magnified it. For example, a patient who has psychosocial problems stemming mainly from depressive symptoms and an underlying personality disorder who then uses alcohol combined with cocaine becoming dysphoric with feelings of hopelessness and abandonment and considers carrying out a suicidal act as a result, is considered a case of Inadequate Coping (IC). However, when a patient has a drug induced hallucinatory experience relating to suicidality, the entrapment is primarily induced – but not magnified – by the use of substances and can be considered Perceptual Disintegration (PDC). Based on this understanding the description for the criteria for PD and IC were adjusted and SUICIDI-tool version 3.2 was established (19).

### 2.3 Statistical analyses

Intraclass Correlation Coefficients (ICCs) with 95% Confidence Intervals (CIs) were calculated using SPSS version 27 (IBM Corp., Chicago, IL). Interrater reliability was assessed using a two-way mixed-effects ICC model with absolute agreement, in line with established guidelines for ICC selection (17). This model was chosen as all cases were rated by the same fixed set of raters and the aim was to quantify agreement within this rater group rather than to generalize reliability estimates to other raters. ICC values < 0.50 indicate poor reliability, 0.50–0.75 moderate reliability, 0.75–0.90 good reliability, and >0.90 indicate excellent reliability (17).

### 2.4 Patient and public involvement

The model validated in this study was developed in close collaboration with three experiential experts in the field of suicidality. One expert contributed insights based on personal lived experience, while the other two drew from their experiences as family members of individuals affected by suicidality. These experts also reviewed and contributed to the final version of the article and are co-authors.

### 2.5 Ethical considerations

The Mental Health Institute Parnassia Group’s research committee approved the study protocol. The Medical Research Ethics Committee Leiden-The Hague-Delft approved the study and data anonymization (reference number (G21.021/PV/pv), waiving the requirement for written informed consent as described previously (15).

## 3 Results

All raters were able to assign cases to subtypes using the SUICIDI-3.2.

**Table 1** describes demographic and clinical features of the cases. Depressive disorders and personality disorders were most common among patients. **Table 2** shows the ICC’s for absolute and dimensional Type Agreement. For absolute Type Agreement (aTA), ICCs demonstrated excellent reliability for Perceptual Disintegration (PD) (0.96) and Primary Depressive Cognition (PDC) (0.92), and good reliability for Psychosocial Turmoil (PT) (0.83) and Inadequate Coping/Communication (IC) (0.89). The lower ICC for PT and its wider 95% confidence interval indicate that PT is most likely associated with other subtypes. This is in line with clinical findings that suicidality often arises from impactful life events (see **Table 2**). In 13.3% of the cases, PD was selected; PDC was chosen in 42.0% of cases; PT in 21.8%; and IC in 22.9%.

**Table 1:** Demographic, clinical and treatment characteristics of cases (n=75).

Characteristic	n (%) *
Female gender	41 (55)
Mean age (SD)	42 (18)
Dutch ethnicity	47(63)
Suicide attempt	21 (28)
Receive actual treatment	16 (21)
IHT** or admission	28 (37)
Assessed outside office hours	38 (51)
Primary DSM-5 classification	
Major depressive disorder	30 (40)
Alcohol/substance abuse	7 (9)
Anxiety disorder (including PTSD***)	7 (9)
Psychotic disorder	5 (7)
Bipolar disorder	4 (5)
Personality disorder	11 (15)
Other disorder (including deferred)	11 (17)

* Unless otherwise specified.

** Intensive Home treatment.

*** Post traumatic stress disorder.

**Table 2:** Intraclass Correlation Coefficient values examining the reliability of subtype agreement among raters (n=6).

Type Agreement	ICC*	95% CI**	Value	Crohnbach-Alpha
absolute Type Agreement (aTA)				
Perceptual Disintegration (PD)	0.96	0.94-0.97	24.85	0·960
Primary Depressive Cognition (PDC)	0.92	0.89-0.94	12.84	0·922
Psychosocial Turmoil (PT)	0.83	0.76-0.89	6.45	0·845
Inadequate Coping (IC)	0.89	0.85-0.93	9.51	0·895
dimensional Type Agreement (dTA)				
Perceptual Disintegration (PD)	0.97	0.96-0.98	36.70	0·947
Primary Depressive Cognition (PDC)	0.95	0.93-0.97	23.30	0·972
Psychosocial Turmoil (PT)	0.88	0.83-0.92	10.11	0·957
Inadequate Coping (IC)	0.92	0.89-0.95	13.68	0·883

* Intraclass correlation coefficient.

** Confidence interval lower bound – upper bound.

For dimensional type agreement (dTA), ICCs were excellent for PD (0.97), PDC (0.95), and IC (0.92), and good for PT (0.88) (see also **Table 2**).

Rater background (psychiatrist vs. nurse) did not significantly affect ICC outcomes, suggesting that the model’s subtypes are simultaneously recognized by clinicians of different professional backgrounds. Subtypes tended to be associated with the primary DSM-V classification, however this is not a consistent finding (see **Supplementary Table 1**).

## 4 Discussion

### 4.1 Main findings

The hypothesized 4-type Model of Entrapment ((h)4ME) describing subtypes of suicidality was developed in clinical practice and is grounded on a theoretical dimensional approach of psychopathology and personality (12, 13, 18). In the current study, the usability and feasibility of the (h)4ME were examined. The results demonstrate high Intraclass Correlation Coefficients (ICC)-values for both absolute (aTA) and dimensional Type Agreement (dTA), implicating good to excellent reliability (17) of ratings for all subtypes (see **Table 2**).

Subtypes can be considered independently of DSM-5 classifications, supporting the view that although suicidality almost always occurs alongside mental disorder, suicidality may manifest differently across individuals regardless of DSM-5 classifications.

This study meets the appeal to consider suicidality as a clinical entity separate from DSM-5 classifications, and contributes to the call for gaining in-depth knowledge on underlying mechanisms leading to suicidality (20).

### 4.2 Comparison with previous research

Similar findings were shown in a pilot in which the usability of an earlier version of the SUICIDI-tool was examined in a smaller sample of cases (n = 25) (15). However, compared to outcomes in the pilot study, a higher proportion of cases were assigned to PDC, and fewer cases to IC. It is possible that this is a result of the revisions made to the SUICIDI tool prior to the current study (15). The differences, however, in the distribution of subtypes (see **Table 2**), are more evident in type PT and IC than in type PD and PDC. This supports the notion that type PD and PDC represent temporary ‘states of being’ whereas type PT and IC likely refer to coping strategies (‘traits’).

### 4.3 Limitations

Potential source bias should be considered when interpreting the findings. The consultation letters used for rating were produced within a clinical setting closely associated with the development of the model and were written by junior doctors and mental health nurses under the clinical supervision of the first author, who was also involved in the development of the SUICIDI framework. This may have influenced the framing or emphasis of suicidal presentations in the letters, potentially leading to greater conceptual alignment between raters and thereby inflating interrater agreement. Although such supervision is routine in clinical practice, future studies should examine the reliability of the model using independently generated clinical data.

Raters were required to assign cases to one or more subtypes as defined in the model and the SUICIDI-3.2. This does not preclude the possibility that more subtypes may exist and may simultaneously co-occur with the currently identified subtypes. This limitation may have resulted in an overestimation of the interrater agreement. It is conceivable that the current subtypes could be further subdivided. This refinement may lead to a reduction of interrater agreement due to an increased likelihood of variability in ratings.

Cases can cover various subtypes, but it is unclear how coverage is distributed over subtypes (12). It is possible that certain subtypes are composed of several mixtures. Thus, there may be overlap between different forms of entrapment. Therefore, a dimensional rather than an absolute approach for subtyping is preferred.

The model was tested in the context of emergency psychiatric care. This concerns the model’s developers, subjects, and raters and may have led to overestimated interrater agreement. Suicidal patients requiring assessment by emergency services likely differ from patients presenting in outpatient community services. At psychiatric emergency services, the focus on suicidality is undisputed, even in patients who are not referred due to suicidality. In outpatient (mental) health services or in the general population, suicidality of a certain subtype may remain more often unnoticed whereas suicidality of another subtype is more likely expressed. This may result in other distributions of subtypes in various populations and contexts. Type PDC and IC for instance, represent more persistent underlying suicidality whereas type PD and PT represent more acute suicidality of people who may be reluctant to seek help.

As last limitation we acknowledge that the high level of expertise among raters may limit the generalizability of the findings to less experienced clinicians or other care settings. This underscores the importance of adequate training in the application of the model, and future work should assess its usability and reliability across varying levels of clinical experience.

### 4.4 Implications for clinical practice

A key starting point for the model’s development was ensuring the model’s applicability and recognizability for healthcare professionals in clinical practice. Many efforts are made to reduce suicidality and psychotherapeutic interventions show promising results (23). By differentiating suicidality, we expect that it may be easier to implement targeted interventions for different subtype groups.

The current model will hopefully make it easier to recognize high risk patients for relatives and professionals who do not have extensive knowledge of suicide risk assessment. It may lead to a better understanding of the efficacy of treatment and counseling options. This might stimulate effective use of mental health care and other means. Direct interventions developed for the treatment of suicidality, such as Cognitive Behavioural Therapy for Suicide Prevention (CBT-SP), Collaborative Assessment and Management of Suicidality (CAMS), Dialectical Behavioral Therapy (DBT) for Suicidal Patients (DBT-SP) (24), and the Attempted Suicide Short Intervention Program (ASSIP), may be more appropriately tailored to address specific subtypes (13).

With respect to the co-occurrence of subtypes, identifying the most apparent points of convergence could enhance the effectiveness of interventions in individual cases.

### 4.5 Implications for future research

Future studies could identify and incorporate additional subtypes into both the model and the SUICIDI-3.2 tool. Social and cultural differences in suicidality may be better understood in terms of variation in the prevalence and clinical presentation. For example, psychosocial turmoil may be encountered less frequently within mental health services overall, including emergency psychiatric settings, whereas inadequate communication may be relatively overrepresented due to its more readily identifiable presentation. It is of interest to further examine the model’s usability and feasibility in subject samples other than adult psychiatric emergency populations, for example chronically suicidal patients, people with addiction problems or people who do not receive specific mental health care. Specific groups of patients should be involved, such as elderly and youngsters dealing with life-stage related problems.

Also, several types of raters should be involved, both from within and outside (mental) health care, such as general practitioners, clinical nurse specialists, psychologists, counselors, and social workers. Future research might also offer insight into possible overlap or co-occurrence between suicidal subtypes and their stability over time. It may also suggest the presence of additional subtypes, such as in elderly who suffer from more existential issues and somatic complaints without overt mental disorder.

Currently, a replication study on the model’s reliability and feasibility is being conducted at Mental Health Institute GGZ Friesland. Raters are six psychologists, who independently allocate 75 cases according to the method of the current study.

From a clinical perspective, further subdifferentiation of type PD could involve the difference between mood-congruent and mood-incongruent presentations. Additionally, PDC might be classified as primary affective suffering or existential distress that does not meet the criteria for a depressive disorder but is instead driven by existential distress. PT could also be divided into internalizing and externalizing suicidality, while IC might be further divided into deliberate and non-deliberate suicidality, among other potential categories.

At this moment, there is no clear and unambiguous evidence for substances to be linked to suicidality. The differentiation in subtypes may contribute to the refinement of pathways leading to suicidality in substance abusers. The enhancement of differentiation of groups could signify the emergence of distinct cultural, economic, psychological, psychosocial, biological dysregulation, or underlying biological/genetic vulnerabilities (25–27).

A previous network study on the pathway of suicidality demonstrated that suicidal ideation is highly individualized. Improved differentiation may provide a better understanding of the underlying factors (28). Finally, psychological autopsy studies of completed suicide may reveal how subtypes are distributed and in what context (29). This may enhance efforts and research into the effectiveness of targeted interventions for suicide prevention.

## 5 Conclusions

The findings suggest that distinguishing four suicidal subtypes is feasible and clinically interpretable, warranting further investigation in future studies. The model demonstrates feasibility and usability in clinical practice, although further research is needed to confirm its generalizability and validity. Clinical adoption of the suicidal subtype model may provide mental health professionals with a structured approach to understand and manage suicidality, promoting tailored interventions and enhancing patient care.

## Data Availability

The raw data supporting the conclusions of this article will be made available by the authors, without undue reservation.
