# Supplementary material for: Identifying subtypes of suicidality: a second larger consensus study in emergency clinical psychiatric practice
Source: Front Psychiatry. 2026 Mar 5;17:1751407. doi: 10.3389/fpsyt.2026.1751407 (PMC12999859; doi:10.3389/fpsyt.2026.1751407)
Supplement: Supplementary file 1 [file Table1.docx]

**Appendix A: Supplementary:** **Identifying Subtypes of Suicidality: A Second Validation Study in Emergency Clinical Psychiatric Practice**

**Supplementary table a:** Assignments of cases (n=75) to absolute subtypes according to raters (n=6) and the primary classification of each case (with the second or other classification between brackets). Dark green (100%): All 6 raters agree. Light green (>83.3%): Only one rater disagrees (5/6 raters agree). Orange (>66.6%): A majority (4/6 raters) agreeing. Red (≤50%): If 3 or fewer raters agree. In 6.7% of cases, two subtypes were selected in equal numbers and are both red.

| **Case**  **no** | **Type I**  **(PD)**  **n** | **Type 2**  **(PDC)**  **n** | **Type 3**  **(PT)**  **N** | **Type 4**  **(IC)**  **N** | **Raters**  **N = 6** | ***Classification***  ***(other secondary classification(s), x and - is none)*** |
| --- | --- | --- | --- | --- | --- | --- |
| **26** | 4 | 2 |  |  |  | *PSD (DD)* |
| **27** |  | 5 |  | 1 |  | *PSD (-)* |
| **28** | 5 | 1 |  |  |  | *DD (-)* |
| **29** |  | 4 |  | 2 |  | *DD (AD)* |
| **30** |  | 4 | 1 | 1 |  | *DD (PSD, AS)* |
| **31** |  |  | 2 | 4 |  | *DD (PSD)* |
| **32** |  | 6 |  |  |  | *DD (-)* |
| **33** |  |  | 6 |  |  | *PSD (AS)* |
| **34** | 1 | 5 |  |  |  | *AD (DD)* |
| **35** | 6 |  |  |  |  | *PD (-)* |
| **36** |  | 3 | 3 |  |  | *DD (ADD)* |
| **37** |  | 2 | 3 | 1 |  | *DD (-)* |
| **38** |  |  | 6 |  |  | *PD (DD, AS)* |
| **39** | 1 | 5 |  |  |  | *ADD (LI)* |
| **40** | 6 |  |  |  |  | *PD (AS)* |
| **41** |  | 5 | 1 |  |  | *DD (AD)* |
| **42** |  | 6 |  |  |  | *DD (-)* |
| **43** |  | 3 | 3 |  |  | *DD (-)* |
| **44** |  |  | 6 |  |  | *AS (-)* |
| **45** | 6 |  |  |  |  | *BD (-)* |
| **46** |  | 6 |  |  |  | *DD (-)* |
| **47** | 1 | 5 |  |  |  | *DD (-)* |
| **48** |  |  |  | 6 |  | *PSD (-)* |
| **49** |  | 1 |  | 5 |  | *ASD (PSD,* |
| **50** |  | 1 |  | 5 |  | *PSD (PTSD, DD)* |
| **51** |  | 3 | 3 |  |  | *DD (-)* |
| **52** |  | 5 | 1 |  |  | *DD (PTSD)* |
| **53** |  |  | 5 | 1 |  | *ASD (DD)* |
| **54** | 6 |  |  |  |  | *PD (DD)* |
| **55** |  | 5 | 1 |  |  | *DD (-)* |
| **56** |  |  | 3 | 3 |  | *AS (-)* |
| **57** | 1 | 5 |  |  |  | *DD (-)* |
| **58** |  |  | 5 | 1 |  | *AS (-)* |
| **59** | 2 |  |  | 4 |  | *PD (AS, ASD, PSD)* |
| **60** |  |  | 2 | 4 |  | *DD (LI)* |
| **61** |  | 5 |  | 1 |  | *PTSD (AD)* |
| **62** |  | 6 |  |  |  | *DD (-)* |
| **63** |  | 5 | 1 |  |  | *AD (DD)* |
| **64** |  | 6 |  |  |  | *PTSD (AD)* |
| **65** |  | 1 | 3 | 2 |  | *DD (-)* |
| **66** |  | 5 |  | 1 |  | *DD (PSD)* |
| **67** |  | 4 | 2 |  |  | *DD (-)* |
| **68** |  |  |  | 6 |  | *PSD (PTSD, AS)* |
| **69** |  | 6 |  |  |  | *BD (-)* |
| **70** |  | 6 |  |  |  | *DD (-)* |
| **71** |  | 6 |  |  |  | *DD (PTSD)* |
| **72** | 1 | 5 |  |  |  | *DD (-)* |
| **73** |  | 5 | 1 |  |  | *DD (-)* |
| **74** |  | 2 |  | 4 |  | *PD (LI)* |
| **75** |  |  | 2 | 4 |  | *AS (ADD)* |
| **76** |  | 6 |  |  |  | *DD (AS, PSD)* |
| **77** |  | 5 | 1 |  |  | *DD (-)* |
| **78** |  | 5 |  | 1 |  | *DD (-)* |
| **79** |  |  | 3 | 3 |  | *PSD* |
| **80** |  |  |  | 6 |  | *AS (PTSD, PSD)* |
| **81** |  |  |  | 6 |  | *AS (DD)* |
| **82** |  | 6 |  |  |  | *DD (-)* |
| **83** |  | 1 | 3 | 2 |  | *AS (PTSD)* |
| **84** |  |  |  | 6 |  | *PSD (ASD)* |
| **85** |  | 6 |  |  |  | *DD (-)* |
| **86** | 6 |  |  |  |  | *PD (DD, LI)* |
| **87** |  | 1 | 1 | 4 |  | *DD (PTSD, LI)* |
| **88** |  |  | 6 |  |  | *PTSD (-)* |
| **89** |  |  | 6 |  |  | *PSD (-)* |
| **90** | 2 | 4 |  |  |  | *PD (-)* |
| **91** |  | 4 | 2 |  |  | *DD (-)* |
| **92** |  | 1 | 3 | 2 |  | *DD (ASD)* |
| **93** |  |  | 5 | 1 |  | *AS (-)* |
| **94** |  |  | 5 | 1 |  | *AS (PSD)* |
| **95** |  |  |  | 6 |  | *DD (AS, PSD)* |
| **96** |  | 6 |  |  |  | *DD (PTSD)* |
| **97** | 6 |  |  |  |  | *BD (-)* |
| **98** |  |  | 3 | 3 |  | *X (-)* |
| **99** | 6 |  |  |  |  | *PD (-)* |
| **100** |  |  |  | 6 |  | *DD (-)* |
|  |  |  |  |  |  |  |
| **Total** | 60 (13.3%) | 189  (42%) | 98 (21.8%) | 103  (22.9%) | 450 (100%) |  |

*Anxiety Disorder (AD), Attention-Deficit (Hyperactivity) Disorder (ADD), Alcohol/Substance abuse (AS), Autism Spectrum Disorder (ASD), Bipolar Disorder (BD), Depressive Disorder (DD),Eating Disorder (ED), PerSonality Disorder (PSD), Post-Traumatic Stress Disorder (PTSD), Psychotic Disorder (PD), Low IQ* (LI).
